# Naphthalimide-based fluorescent polymeric probe: a dual-phase sensor for formaldehyde detection

**DOI:** 10.1080/14686996.2025.2469493

**Published:** 2025-02-21

**Authors:** Subhadip Roy, Swagata Pan, Swaminathan Sivaram, Priyadarsi De

**Affiliations:** a Polymer Research Centre and Centre for Advanced Functional Materials, Department of Chemical Sciences, Indian Institute of Science Education and Research Kolkata, Nadia, West Bengal, India; b Indian Institute of Science Education and Research Pune, Pune, India

**Keywords:** Formaldehyde, polymeric probe, fluorometric sensing, Schiff base formation, aqueous and vapor phase detection

## Abstract

Formaldehyde (FA) is a common pollutant found indoors and outdoors, posing a significant threat to human health. Therefore, developing sensitive and efficient detection methods for FA is essential for environmental monitoring and protecting public health. Herein, we report a naphthalimide-conjugated water-soluble polymeric fluorescent probe for the detection of FA in both aqueous and vapor phases using fluorimetric methods. The aromatic amines present in the side chain of the polymer react with FA, forming a Schiff base (imine bond). This imine formation inhibits the photoinduced electron transfer (PET) process within the polymer, leading to a ‘turn-on’ fluorescence under 365 nm UV light. The probe is capable of selectively sensing FA with a detection limit as low as 1.36 nM in aqueous medium. The formation of imine is confirmed for the model reaction between 6-(4-aminophenyl)-2-(4-((4-vinylbenzyl)oxy)phenyl)-1 h-benzo[de]isoquinoline-1,3(2 h)-dione and FA by electrospray ionization mass spectrometry (ESI-MS) and nuclear magnetic resonance (NMR) titration methods. The mechanism behind ‘turn-on’ FA sensing is investigated using density functional theory (DFT) analysis. Additionally, the study demonstrates a facile approach for covalently attaching the polymer on the surface of a filter paper surface *via* ultraviolet (UV) light-induced cross-linking. Such polymer attached paper exhibits FA vapor sensing through changes in fluorescence intensity.

## IMPACT STATEMENT

A naphthalimide-conjugated fluorescent water-soluble polymeric probe is reported for formaldehyde sensing in an aqueous (nanomolar level) phase within a very short time (1 min) with high selectivity. Also, this work demonstrates a facile approach for FA vapor sensing through changes in fluorescence intensity.

## GRAPHICAL ABSTRACT

**Figure:**
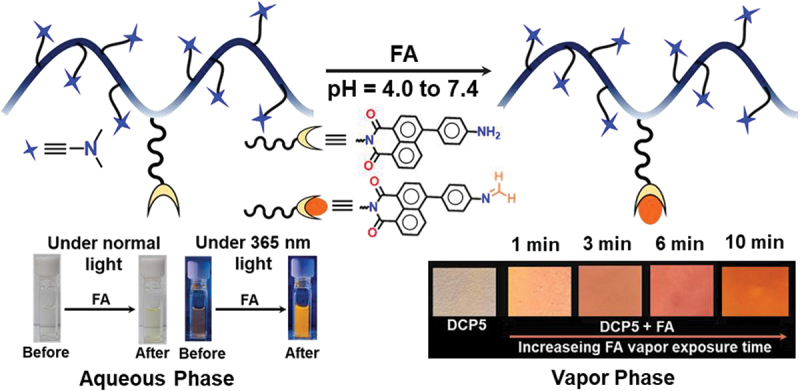

## Introduction

Formaldehyde (FA), one of the most reactive carbonyl species, is widely utilized as a basic raw material in building construction, paints and coatings, furniture making, textile production, and the chemical industry [1]. Formaldehyde production exceeds 20 million tons per annum and has emerged as a major environmental risk since its inhalation can lead to serious health-related concerns [2]. Moreover, FA inhalation promotes the growth of squamous cell carcinomas in rats’ nasal passages and nasopharyngeal cancer in humans [3]. The International Agency for Research on Cancer (IARC) has classified FA as a Group 1 carcinogen for both humans and animals [4]. Typically, 750 ppb is the standard permissible exposure limit set by the Occupational Safety and Health Administration (OSHA), while 20 ppm is considered the highest exposure value, which is immediately dangerous to life or health (IDLH) [5]. Beyond its well-known man-made origins, FA is also produced inside organisms, where it plays a variety of physiological and pathological activities [6]. FA content in biological systems can be influenced by both exogenously delivered and endogenously generated sources. Consequently, elevated cellular FA levels have been associated with a range of chronic disorders, including heart disease, leukemia [7], diabetes [8], Alzheimer’s disease [9], and numerous malignancies.

Recently, significant efforts have been devoted towards the development of formaldehyde sensing methods, including potentiometry [10], mass spectrometry [11], electrochemical analysis [12], etc. Among these, fluorescence-based techniques have emerged as the most promising approach because of their ease of use and high detection sensitivity, drawing widespread interest from the scientific community. To date, numerous small molecule-based fluorescent sensors have been designed for formaldehyde sensing, based on FA amine condensation [13], hydrazine (-NH-NH_2_) condensation [14,15], and aza-Cope rearrangement reaction [16]. However, these small molecular probes contain hydrophobic organic chromophores, resulting in poor water solubility and limiting their applications in aqueous medium and biological environments [17]. Consequently, performing sensing experiments needs volatile and hazardous organic solvents or hybrid aqueous solutions, which significantly reduce their utility in applications [18,19]. Therefore, developing a water-soluble probe with a lower limit of detection (LOD) and rapid sensing capability is essential for precise and quantitative measurement of FA in both biological and environmental milieu.

In this regard, water-soluble fluorescent polymeric probes have emerged as a promising alternative, providing a broad range of potential advantages that overcome the challenges associated with formaldehyde detection [20]. For example, Liu *et al*. designed a polymeric probe *via* the Hantzsch reaction, which detects endogenous FA in living cells [21]. Li and coworkers reported chitosan-based water-soluble polymers for FA detection in various food samples [22]. Also, a porous polymer composite was reported to remove gaseous FA *via* oxime bond formation at room temperature [23]. Recently, our group reported a side-chain tryptophan-based water-soluble polymeric probe to detect FA through a Pictet-Spengler cyclization reaction in an aqueous medium [24]. Although these polymeric probes detected FA in an aqueous medium, their applications to sensing FA in the vapor phase were not explored, possibly because of their long equilibrium time to detect FA quantitatively. Detection of gaseous FA and toxic volatile organic compounds (VOCs) is a crucial frontier in scientific and technological progress [25,26], essential for improving safety, environmental friendliness, and ease of handling [27]. Therefore, the design of sensitive and reliable polymeric sensors for FA vapor has gained much interest due to the demand for real-time monitoring of air quality and workplace safety [28,29]. To address these issues, we aimed to develop a water-soluble fluorescent polymeric probe to detect FA both in the aqueous and vapor phases through fluorogenic response.

To this end, naphthalimide-based water-soluble polymeric probe with amino groups in pendant naphthalimide moieties was synthesized using reversible addition-fragmentation chain transfer (RAFT) polymerization for rapid FA detection in both aqueous and vapor phases. Initially, naphthalimide was introduced as a signal reporting unit and functionalized with an amine group to facilitate binding with formaldehyde. The probe’s design is based on a specific chemical reaction between FA and the aromatic amino group of the polymer, which results in a ‘turn-on’ fluorescence response from the side-chain naphthalimide fluorophores. Therefore, the designed polymeric probe can take advantage of the synergistic effects of many recognition sites in the side chain *via* weak supramolecular interactions to efficiently ‘concentrate’ low amounts of FA pollutants around the polymer’s random coil chains [30]. With this innovative strategy, the addition-elimination reaction between FA and aromatic amino groups of naphthalimide-conjugated polymer is accelerated significantly, leading to a fluorescence enhancement within a minute and increased sensitivity. Furthermore, to make a paper strip-based sensor for FA vapor, benzophenone moiety was incorporated in the side chain of the polymer and cross-linked on filter paper *via* irradiation with ultraviolet (UV) light [31].

## Results and discussion

To develop the polymeric FA-sensor, a naphthalimide-conjugated styrenic monomer (Scheme S1) was synthesized by multistep organic synthesis. Initially, 6-bromo-2-(4-((4-vinylbenzyl)oxy)phenyl)-1 h-benzo[de]isoquinoline-1,3(2 h)-dione (**C2**) was synthesized [32], and subsequently reacted with 4-amino boronic acid *via* Suzuki-Miyaura coupling reaction to develop aromatic amine-conjugated 6-(4-aminophenyl)-2-(4-((4-vinylbenzyl)oxy)phenyl)-1 h-benzo[de]isoquinoline-1,3(2 h)-dione (**C3**). Finally, the desired styrenic monomer, *tert*-butyl (4-(1,3-dioxo-2-(4-((4-vinylbenzyl)oxy)phenyl)-2,3-dihydro-1 h-benzo[de]isoquinolin-6-yl)phenyl)carbamate (NDIST), was prepared by protecting the free aromatic amine group of **C3**. **C1**, **C2**, and **C3** were characterized using ^1^H NMR spectroscopy and electrospray ionization mass spectrometry (ESI-MS) (Figure S1–S4). The final monomer **NDIST** was characterized using ^1^H NMR, ESI-MS, attenuated total reflection Fourier transform infrared spectroscopy (ATR-FTIR), and ^13^C NMR spectroscopy (Figure S5–S8).

**Figure 1.**
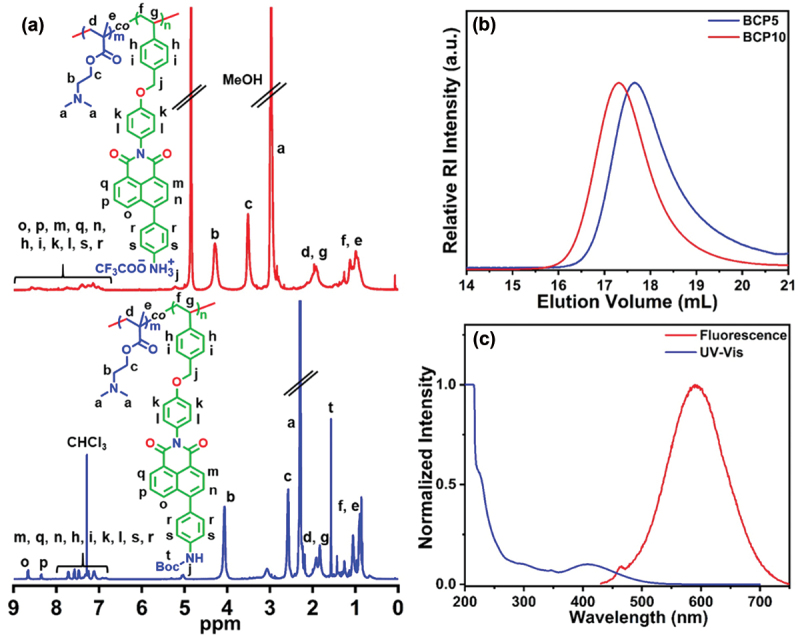
(a) ^1^H NMR spectra of **BCP5** recorded in CDCl_3_ (bottom) and **DCP5** recorded in MeOH-*d*_4_ (top). (b) SEC refractive index (RI) traces of BCP5 and BCP10 were recorded in DMF at 40°C. (c) Normalized UV-Vis and fluorescence spectra of deprotected copolymer (0.25 mg/mL aqueous solution of **DCP5**).

**Scheme 1.**
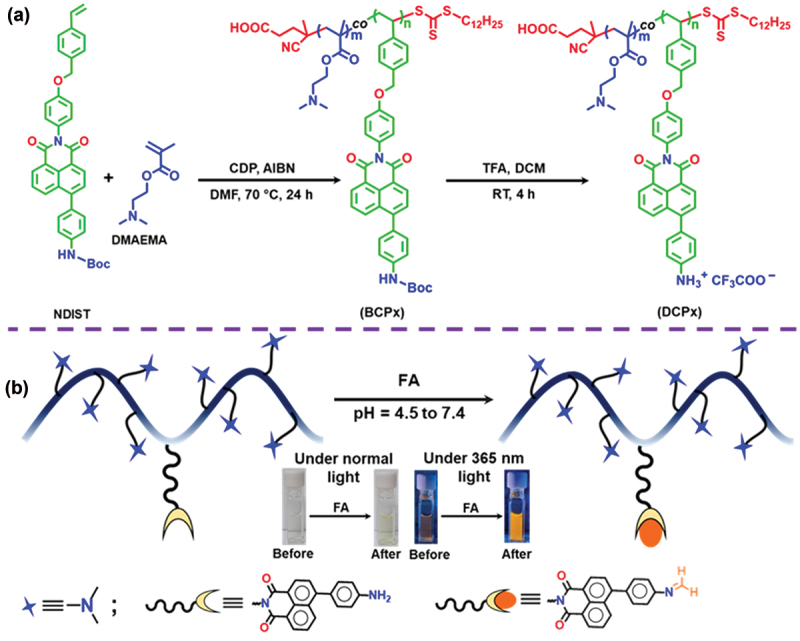
(a) synthetic route for the preparation of naphthalimide-conjugated polymeric probe. (b) Schematic representation of FA sensing mechanism with the naphthalimide-conjugated polymeric probe.

To prepare a water-soluble FA-sensing polymeric probe with controlled molecular weight and narrow dispersity (*Ð*), RAFT copolymerization was employed using water-soluble 2-(dimethylamino)ethyl methacrylate (DMAEMA) and hydrophobic NDIST monomers, in the presence of 4-cyano-(dodecylsulfanylthiocarbonyl)sulfanylpentanoic acid (CDP) as a RAFT agent and 2,2′-azobis(isobutyronitrile) (AIBN) as a radical source, in *N,N*-dimethylformamide (DMF) at 70°C (Scheme 1(a)). Two copolymers, **BCP5** and **BCP10** were synthesized by varying the molar ratio of DMAEMA and NDIST, keeping the molar ratio of [CDP]:[AIBN] constant at 1:0.2 (Table S1). The non-appearance of vinyl proton peaks between 5.2–6.7 ppm in the ^1^H NMR spectrum (Figure 1(a), bottom) confirmed the successful removal of monomers in the final copolymers. The composition of DMAEMA and NDIST in the copolymers was calculated by comparing the peak intensity of the benzylic protons of NDIST moiety at 5.15–5.20 ppm and the dimethyl protons (-N(CH_3_)_2_) peak from DMAEMA unit at 2.3 ppm (Table S1). The number average degrees of polymerization (*DP*) for both DMAEMA and NDIST in the copolymer was determined by comparing the integration values of chain end protons [-CH_2_-(CH_2_)_9_-CH_3_] of CDP at 0.8–0.9 ppm to the characteristic peaks of -N(CH_3_)_2_ protons at 2.3 ppm from DMAEMA units and benzylic protons from NDIST at 5.15–5.20 ppm, respectively. The calculated *DP*_DMAEMA_ and *DP*_NDIST_ of **BCP5** were 80 and 4, respectively. The number average molecular weight of the copolymers was determined from ^1^H NMR analysis (*M*_n,NMR_) using the formula (Table S1): *M*_n,NMR_ = [(*DP*_DMAEMA_ × *MW*_DMAEMA_) + (*DP*_NDIST_ × *MW*_NDIST_) + molecular weight of CDP], where *MW* represents the molecular weight of the two monomers. The size exclusion chromatography (SEC) was performed to determine the *Ð* and number average molecular weight (*M*_n,SEC)_ of copolymers (Figure 1(b) and Table S1). The theoretical molecular weights (*M*_n,theo_) of the copolymers were also determined and results are shown in Table **S1**. The *M*_n,theo_, *M*_n,NMR_ and *M*_n,SEC_ values in Table **S1** match reasonably well with each other, indicating the controlled nature of RAFT copolymerization of DMAEMA and NDIST. Finally, to prepare the desired naphthalimide-functionalized polymeric probe with pendant aromatic amine groups, *tert-*butyloxycarbonyl (Boc) groups of both **BCP5** and **BCP10** were deprotected using trifluoroacetic acid (TFA) in dichloromethane (DCM) at room temperature. The resulting deprotected copolymers were labelled as **DCP5** and **DCP10**, respectively. The successful cleavage of Boc groups was verified by the disappearance of the characteristic protons peak of Boc group at ~ 1.4 ppm range in the ^1^H NMR spectrum of **DCP5** (Figure 1(a), top). Furthermore, both the **DCP5** and **DCP10** copolymers were nicely soluble in water.

**Figure 2.**
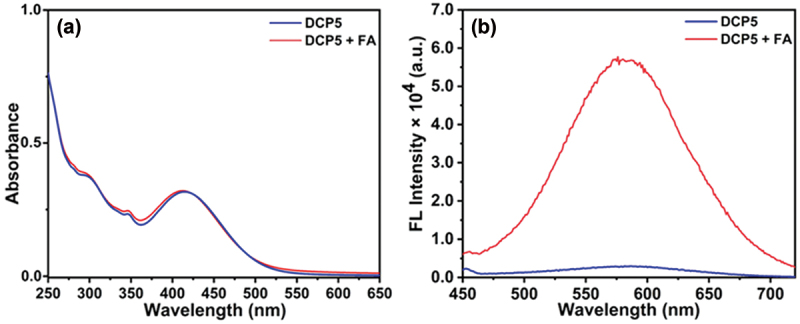
(a) UV-Vis and (b) fluorescence spectra of aqueous **DCP5** solutions (0.25 mg/mL) in the absence and presence of 100 nM of FA.

With water-soluble aromatic amine-conjugated naphthalimide-based polymers in hand, we sought to investigate their performance towards FA sensing in an aqueous medium. Initially, the UV-Vis and fluorescence analyses were carried out on both polymers in water. **DCP5** exhibited two absorption maxima at 280 nm and 420 nm in an aqueous medium, referrers to the π-π* and n-π* transitions for the naphthalimide unit (Figure 1(c)). Although both polymers were water-soluble, **DCP10** exhibited self-assembly in an aqueous medium due to the higher content of hydrophobic NDIST units (data not shown here), which could potentially increase the response time. Our primary focus is on rapid FA detection in aqueous media, so the self-assembly characteristics of **DCP10** were not extensively studied. Therefore, **DCP5** was chosen for further studies to prevent such complications. As shown in Figure 2(a), the addition of 100 nM FA to the aqueous solution of **DCP5** resulted in a slight blue shift (8 nm) in the UV-Vis spectrum. A subtle color change from colorless to light yellow was also observed under normal light. However, **DCP5** exhibited a significant fluorogenic response upon FA treatment, where the polymer displayed an emission maximum at 580 nm when excited at 420 nm (Figure 2(b)).

**Figure 3.**
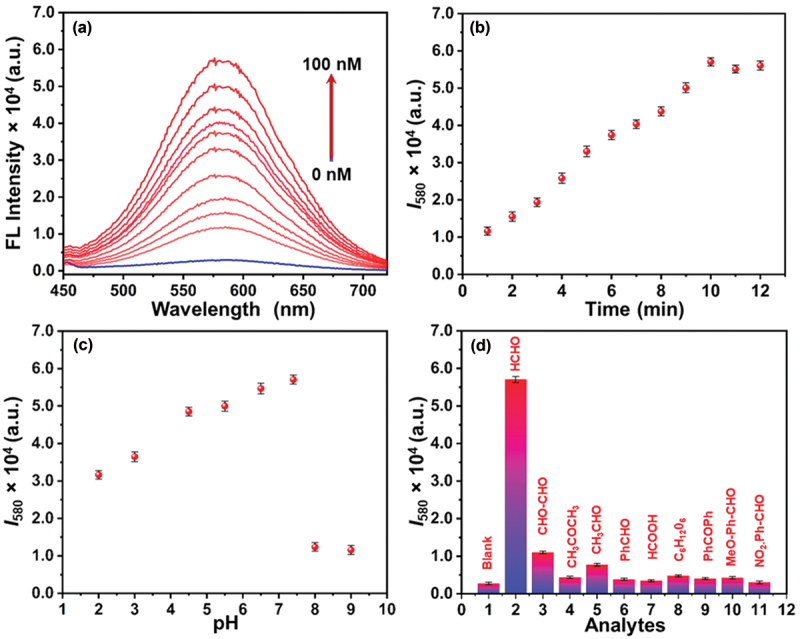
(a) the emission spectra of **DCP5** solutions (0.25 mg/mL) in the presence of various concentrations of FA, measured 1 min after FA addition. (b) Time-dependent emission intensity change of **DCP5** solutions at 580 nm with 100 nM of FA. (c) Fluorescence intensity of the aqueous **DCP5** solutions (0.25 mg/mL) at different pH levels after reaction with 100 nM FA. (d) Bar graph illustrating the emission intensities of **DCP5** (0.25 mg/mL) at 580 nm in the presence of FA and other analytes (measured after 10 min).

The presence of an aromatic amine group in the naphthalimide unit quenched the strong fluorescence of naphthalimide moiety *via* the photoinduced electron transfer (PET) process [33]. However, upon the addition of an increasing amount of FA (10 to 100 nM), the fluorescent intensity at 580 nm gradually increased and reached the maximum in the presence of 100 nM FA (Figure 3(a)). The noticeable increment in fluorescence intensity indicated the significant halt of the PET process during the fast Schiff base reaction between FA and the **DCP5**. This shows that the polymeric probe can sense a very low concentration of FA with outstanding fluorescence enhancement. Although the FA sensing mechanism of the amine-functionalized **DCP5** is based on imine formation reaction processes, it is imperative to study the kinetic profiles after FA addition. Hence, the time-dependent fluorescence behavior of the polymeric probe towards FA was examined. The fluorescence experiments were performed within cuvettes in a meticulously controlled manner. Fluorescence spectra of the aqueous solution of the polymeric probe were recorded at different time intervals upon the addition of 100 nM FA. As demonstrated in Figure 3(b), the polymeric probe showed a quick response within just 1 min, with the fluorescence emission intensity at 580 nm gradually increased and saturated within 10 min. The limit of detection (LOD) for the **DCP5** towards FA was determined as 1.36 nM (Figure S9) [34]. Additionally, the absolute quantum yield of **DCP5** increased from 1.15% to 3.15% after reaction with FA (Figure S10). Enhancement in the fluorescence intensity does not directly translate to a proportional increase in quantum yield, as other factors are also involved (Inner-filter effects, excited-state interactions, fluorescence lifetime, etc.). In the fluorescence titration experiment with the model compound (**C3**), the addition of an increasing amount of FA (10 to 100 nM) gradually increased the fluorescent intensity at 580 nm (Figure S11).

To explore the ideal sensing condition, the fluorescence response of **DCP5** (10 mm with respect to the functional naphthalimide unit) to FA was studied across a pH range from 2 to 9 (Figure 3(c)). Upon the addition of FA to **DCP5**, an enhancement in fluorescence intensity was observed between pH 2.0 to 7.4, with a remarkable increase in the range of pH 4.5–7.4. These results imply that the reaction between the FA and the aromatic amine groups present in the side chain of the polymer is more efficient under neutral conditions, making it well-suited for standard applications under different conditions [13]. Furthermore, the sensing selectivity (Figure 3(d)) and photostability (Figure S12) of the **DCP5** probe were studied in an aqueous medium. As depicted in Figure 3(d), only a small increase in fluorescence was observed for several potentially interfering analytes, including various common aldehydes and other chemical species, such as 4-methoxy benzaldehyde, 4-nitrobenzaldehyde, benzaldehyde, formic acid, glyoxal, acetone, glucose, benzophenone, and acetaldehyde. These investigations implied the excellent selectivity of the **DCP5** probe towards FA. Moreover, the emission intensities of **DCP5** were nearly constant under specific experimental conditions (irradiation at 420 nm for 30 min), suggesting its photostability under photoirradiation and exposure to atmospheric conditions (Figure S12).

To confirm the FA sensing mechanism of the polymeric probes, we performed ^1^H NMR titration experiments and ESI-MS analysis. Given the challenges of low resolution in polymeric spectra, we utilized **C3** as a model compound to capture the ^1^H NMR titration spectra. This approach provided valuable insights into the interaction between the polymer and FA, allowing us to elucidate the underlying mechanism of sensing. In Figure S13, the stepwise addition of FA leads to the gradual disappearance of the -NH_2_ proton signal at ~ 5.5 ppm, accompanied by the emergence of a new peak at 9.5 ppm, corresponding to the imine (-N=C***H***_2_) group. In addition, a peak at *m/z*  = 509.1913 was found in the ESI-MS analysis of the reaction mixture of **C3** and FA (Figure S14). These results evidenced the formation of Schiff base (imine bond) during the reaction of FA with **DCP5** in an aqueous medium.

**Figure 4.**
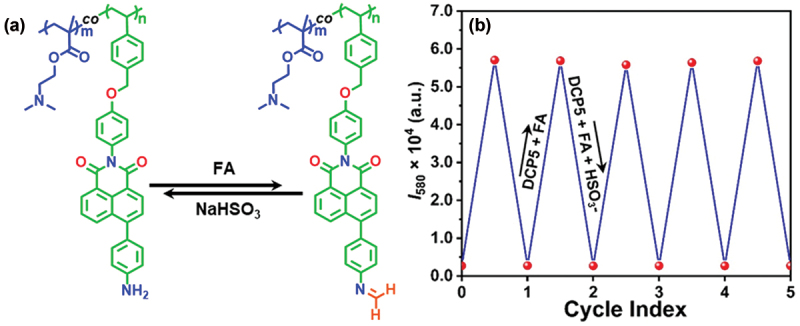
(a) sensing mechanism of the **DCP5** polymeric probe for FA/bisulfite. (b) Reversibility study of **DCP5** between FA and bisulfite.

After confirming the sensitivity and selectivity of **DCP5** for FA, the probe’s reversibility was studied in the presence of bisulfite (HSO_3_^−^) [35]. As shown in Figure 4(a), after the formation of **DCP5**-FA adduct, it was reacted with NaHSO_3_, and the emission intensity returned to its initial level within 3 min, closely aligning with the fluorescence intensity of **DCP5**. This suggested the reversibility of the formation of imine moieties from the aromatic amine groups of **DCP5** and FA. It is envisaged that the HSO_3_^−^ traps the free FA and disrupts the imine adduct present in the polymer, leading to the formation of free amine-containing polymeric probe **DCP5**. The reversibility of **DCP5** was investigated up to five cycles (Figure 4(b)). Thus, in addition to selective and sensitive detection of FA, the **DCP5** copolymer can be effectively reused in the solution phase for multiple cycles.

**Figure 5.**
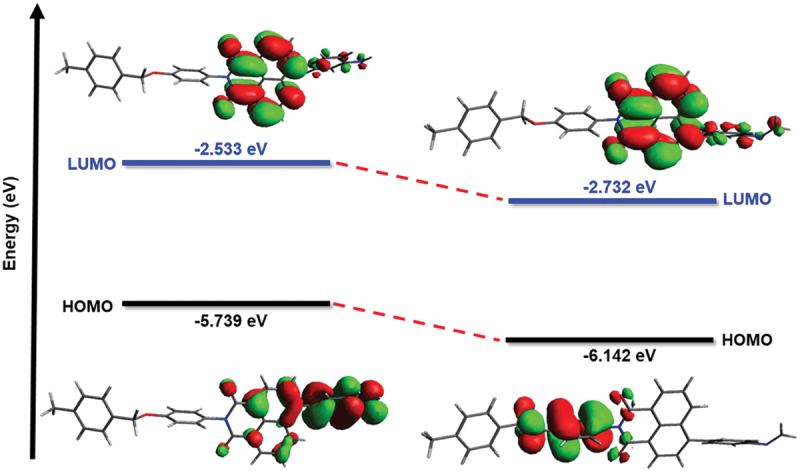
HOMO-LUMO energy difference of the NDIST repeating unit before and after adding FA, calculated using DFT with the B3LYP/6-311 G(d) basis set.

To gain a thorough understanding of the sensing mechanism, density functional theory (DFT) calculation was conducted to evaluate the highest occupied molecular orbital (HOMO) and the lowest unoccupied molecular orbital (LUMO) of the NDIST repeating unit before and after the addition of FA. The HOMO energy of the NDIST (−5.739 eV) was higher than the HOMO energy (−6.142 eV) of the corresponding imine derivative, indicating the PET process was interrupted by the imine formation, leading to a ‘turn-on’ fluorescence response (Figure 5) [36].

**Figure 6.**
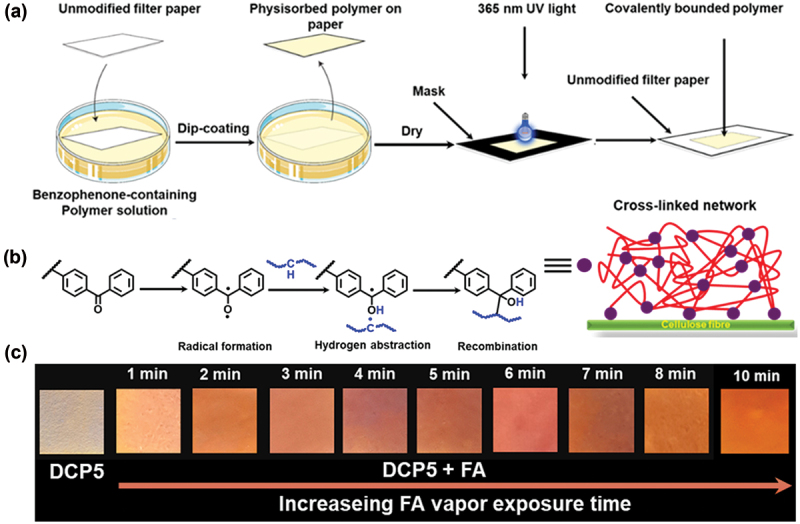
UV-light induced immobilization of benzophenone-containing polymer on the filter paper. (a) General procedure for cross-linking using carbon chart mask. (b) Probable mechanism of light-induced cross-linking of benzophenone moieties. (c) Photographs of **DCP5** coated paper strip before and after the exposure to FA vapor.

To employ the designed polymeric material for practical applications, the vapor phase detection of FA was carried out. It is well-reported that the benzophenone undergoes cross-linking in the presence of UV light (365 nm) [31]. Thus, a terpolymer was prepared using benzophenone-based monomer (BPMA), NDIST, and DMAEMA, following RAFT polymerization (Scheme S2). To attain covalent attachment onto filter paper, the BPMA-containing terpolymer was dissolved in methanol (5 mg/mL). Subsequently, the paper was coated with the polymer solution by dip-coating (submerging for 20 s) and air-dried for 30 min, as shown in Figure 6(a). The polymer-coated filter paper was then placed inside a UV chamber and illuminated with UV light (365 nm, 8 W) to induce cross-linking (Figure 6(b)). To generate well-defined polymer patterns on the paper surface, black chart paper masks were placed on top of the material during the irradiation step. Next, the polymer-coated filter paper was exposed to the FA vapours. As expected, progressively enhanced fluorescence intensity of filter paper was noticed by the naked eye with increasing FA exposure time (0 to 10 min), and fluorescent colors of test strips gradually changed from light yellow to dark orange under 365 nm UV light (Figure 6(c)). These results indicate that the designed polymeric probe could serve as a portable paper-based sensor for detecting FA vapours [37]. The vapour phase sensing experiment was carried out at 27°C. At this temperature, FA concentration was around 0.1 ppm, so the minimum detection limit in the vapour phase is at 0.1 ppm level [2]. Further studies are in progress to quantify the detection level of FA vapors at different humidity/temperature conditions.

## Conclusions

In conclusion, a naphthalimide-conjugated water-soluble copolymer was designed for FA detection in an aqueous solution through fluorimetric analysis. The solution phase fluorescence ‘turn-on’ of **DCP5** was attributed to the formation **DCP5**-FA adduct through Schiff base reaction, in which the ‘turn-on’ fluorescence mechanism operating through the suppression of PET process was supported by DFT calculations. **DCP5** showed excellent selectivity and sensitivity toward FA over other analytes in the pH range of 4.5 to 7.4 with a detection limit of 1.36 nM in an aqueous medium. Interestingly, **DCP5** could be regenerated from the **DCP5**-FA adduct, validating the recyclability and reusability of the polymer. This renders the polymer probe a highly practical and environmentally friendly option for continuous monitoring applications. Preliminary results show that the polymer-based sensor coated on a filter paper could be useful as a point-of-care sensor of FA vapour, wherein the observed visual color change under UV-light exposure could be used as an indicator. Further work is in progress to validate this observation, determine the limits of detection, and demonstrate the usefulness of the method for monitoring FA concentration in ambient air. Thus, the present work not only advances our understanding of the FA sensing mechanism but also paves the way for the development of an effective, efficient, and practical method for the detection of formaldehyde in both the aqueous and vapour phase.

## Supplementary Material

Supplemental Material

## Supporting information

Synthesis and characterization details of NDIST and BPMA, polymers synthesis, photostability study, quantum yield calculation, LOD determination method, and DFT calculation details are provided in supporting information.
