# Supplementary material for: Naphthalimide-based fluorescent polymeric probe: a dual-phase sensor for formaldehyde detection
Source: Sci Technol Adv Mater. 2025 Feb 21;26(1):2469493. doi: 10.1080/14686996.2025.2469493 (PMC11905309; doi:10.1080/14686996.2025.2469493)
Supplement: Supplemental Material [file TSTA_A_2469493_SM5160.docx]

**Supporting Information for**

**Naphthalimide-based fluorescent polymeric probe: A dual-phase sensor for formaldehyde detection**

Subhadip Roy,^1^ Swagata Pan,^1^ Swaminathan Sivaram^2,^* and Priyadarsi De^1,^*

^1^Polymer Research Centre and Centre for Advanced Functional Materials, Department of Chemical Sciences, Indian Institute of Science Education and Research Kolkata, Mohanpur-741246, Nadia, West Bengal, India.

^2^Indian Institute of Science Education and Research Pune, Dr. Homi Bhabha Road, Pune - 411008, India.

*Corresponding authors: emails: s.sivaram@iiserpune.ac.in (SS); p_de@iiserkol.ac.in (PD)

## **Experimental section**

**Materials**. 2-(Dimethylamino)ethyl methacrylate (DMAEMA), 4-vinyl benzyl chloride (4-VBC), 4-aminophenol, sodium *tert*-butoxide (NaO*^t^*Bu), *N*,*N*-dimethylformamide (DMF, 99.9%), formaldehyde (FA, HCHO, 37% in water), phosphate buffered saline (PBS), and di-*tert-*butyl dicarbamate ((Boc)_2_O, 98%) were purchased from Sigma Aldrich. 4-Bromo-1,8-naphthalic anhydride (95%) and palladium-tetrakis(triphenylphosphine) (Pd(PPh_3_)_4_) were bought from BLD Pharmatech Ltd. India. The 2,2′-azobis(isobutyronitrile) (AIBN, 98%, SRL, India) was recrystallized from methanol and used as an initiator during the polymerization. 4-Cyano-(dodecylsulfanylthiocarbonyl)sulfanylpentanoic acid (CDP) was prepared following previous standard literature [1]. Benzophenone-based monomer (BPMA) was prepared following a previous literature procedure [2]. Dialysis membrane with molecular weight cutoff (MWCO) = 6000-8000 g/mol (Spectra/Por®, diameter 14.6 mm, flat width 23 mm, volume/length 1.7 mL/cm) was purchased from Spectrum Laboratories, USA. The NMR solvents CDCl_3_ (99.8% D), MeOH-*d*_4_, and DMSO-*d*_6_ (99% D) were purchased from Cambridge Isotope Laboratories, Inc., USA. Dichloromethane (DCM), chloroform (CHCl_3_), hexane (a mixture of isomers), acetone, methanol (MeOH), ethanol (EtOH), ethyl acetate (EtOAc), trifluoro acetic acid (TFA), diethyl ether, tetrahydrofuran (THF), dimethyl sulfoxide (DMSO), acetonitrile (MeCN), HPLC graded water, potassium carbonate (K_2_CO_3_), sodium sulfate (Na_2_SO_4_), sodium bicarbonate (NaHCO_3_), and hydrochloric acid (HCl) were obtained from Merck, India. THF, hexane, DCM, acetone, EtOAc, and MeOH were purified by following standard protocols [3].

**Instruments and characterizations**

**Nuclear magnetic resonance (NMR).** ^1^H NMR spectroscopic measurements were recorded in either 500 MHz Bruker AvanceIII NMR spectrometer or 400 MHz JEOL ECS NMR spectrometer at 25 °C using tetramethylsilane (TMS) as an internal reference. MeOH-*d*_4,_ DMSO-*d*_6,_ and CDCl_3_ were used as solvents to record the spectra depending on the solubility of the compounds.

**Electrospray ionization mass spectrometry (ESI-MS).** The ESI-MS spectra were measured in Waters Xevo® G2-XS QTof mass spectrometer using positive mode and negative mode electrospray ionization. 1.0 mM concentration of the compounds was prepared in MeCN or MeOH. The solution was filtered *via* a polytetrafluoroethylene (PTFE) syringe filter (0.45 µm) before the experiment.

**UV-Vis spectroscopy.** UV-Vis spectra of the polymers were recorded in a PerkinElmer Lambda 35 spectrophotometer.

**Fluorescence spectroscopy.** The emission spectra of the polymers were measured in a Horiba JobinYvon fluorescence spectrometer (Fluoromax-3, Xe-150 W, 250-900 nm).

**Size exclusion chromatography (SEC).** The number average molar mass (*M*_n,SEC_) and dispersity (*Ð*) of the polymers were determined using SEC in DMF with 0.8 mL/min flow rate at 40 ℃. The instrument contains a Waters 1515 HPLC pump, a Waters 2414 refractive index (RI) detector, two PolarGel-M analytical columns (300 × 7.5 mm), and one PolarGel-M guard column (50 × 7.5 mm). The calibration curve of the instrument was prepared using poly(methyl methacrylate) (PMMA) standards from Agilent technologies. Typically, the polymers were dissolved in DMF (1 mg/mL) and passed through a 0.45 µm PTFE syringe filter before the experiment.

**Attenuated total reflection Fourier transform infrared spectroscopy (ATR-FTIR)**. ATR-FTIR spectroscopy was used to record the bond stretching frequency of samples in the solid state using a Bruker Alpha Platinum-ATR instrument.

**Synthesis of 4-((4-vinylbenzyl)oxy)aniline (C1).** The **C1** was synthesized following the previous literature [4]. Typically, 4-aminophenol (2.5 g, 20.8 mmol) was taken in a 250 mL round-bottom flask and dissolved in 65 mL of DMF. Then, NaO*^t^*Bu (2.2 g, 22.8 mmol) was added to the solution, and after 5 min the flask was kept at 90 ℃. 4-Vinyl benzyl chloride (3.2 g, 20.8 mmol) was added dropwise to the reaction mixture after 10 min of stirring. The completion of the reaction was monitored by thin-layer chromatography. Next, the solution was diluted with 500 mL of DCM. This organic layer was washed with ice-cold water (4 × 200 mL) and brine solution (200 mL). The organic layer was collected and dried over anhydrous Na_2_SO_4_ and purified the compound by column chromatography with 20-25% (v/v) EtOAc in hexane mixture. Yield = 70%. **C1** was characterized using ^1^H NMR spectroscopy. ^1^H NMR (400 MHz, CDCl_3_, *δ* ppm): 7.40 (q, *J* = 8.2 Hz, 4H), 6.84–6.78 (m, 2H), 6.72 (d, *J* = 17.6 Hz, 1H), 6.67–6.60 (m, 2H), 5.76 (d, *J* = 17.6 Hz, 1H), 5.25 (d, *J* = 10.9 Hz, 1H), 4.98 (s, 2H), 3.46 (broad, 2H).

**Synthesis of 6-bromo-2-(4-((4-vinylbenzyl)oxy)phenyl)-1H-benzo[de]isoquinoline-1,3(2H)-dione (C2).** 4-bromo-1,8-naphthalic anhydride (2.4 g, 8.9 mmol) was taken in a 100 mL round-bottom flask and 50 mL of EtOH was added. The flask was kept at 70 ℃ and Et_3_N (1.8 g, 8.9 mmol) was added to the solution. After 15 min of stirring, **C1** (2.0 g, 8.9 mmol) was added to the reaction mixture. The reaction condition was maintained overnight, resulting in the formation of a white precipitate. The reaction mixture was then cooled and the precipitate was filtered and washed with EtOH. The white color compound was dried under vacuum at 45 ℃ overnight. Yield = 75%. **C2** was characterized by ^1^H NMR analysis. ^1^H NMR (500 MHz, DMSO-*d*_6_, *δ* ppm): 8.56 (dd, *J* = 10.4, 7.8 Hz, 2H), 8.30 (d, *J* = 7.9 Hz, 1H), 8.22 (d, *J* = 7.9 Hz, 1H), 8.06–7.95 (m, 1H), 7.53–7.40 (m, 4H), 7.26 (d, *J* = 9.0 Hz, 2H), 7.10 (d, *J* = 8.8 Hz, 2H), 6.73 (dd, *J* = 17.7, 11.0 Hz, 1H), 5.82 (d, *J* = 17.7 Hz, 1H), 5.25 (d, *J* = 10.9 Hz, 1H), 5.14 (s, 2H).

**Synthesis of 6-(4-aminophenyl)-2-(4-((4-vinylbenzyl)oxy)phenyl)-1H-benzo[de]isoquinoline-1,3(2H)-dione (C3).** **C2** (500.0 mg, 1.1 mmol), 4-amino boronic acid (215.4 mg, 1.2 mmol), and K_2_CO_3_ (286.1 mg, 2.1 mmol) were taken in a sealed tube. Next, 7 mL DMF and 2 mL water were added and purged with dry N_2_ for 10 min. After that, Pd(PPh_3_)_4_ (60.0 mg, 51.0 μmol) was added to the reaction mixture. The sealed tube was then placed on a preheated oil bath at 90 ℃ for 18 h. After completion of the reaction, the solution was diluted with 200 mL of DCM. This organic layer was washed with ice water (5 × 200 mL) and brine solution (200 mL). The organic layer was passed through anhydrous Na_2_SO_4_ and purified by column chromatography with 50-60% (v/v) EtOAc in hexane mixture. Yield = 65%. **C3** was characterized by ^1^H NMR and ESI-MS spectroscopy. ^1^H NMR (500 MHz, DMSO-*d*_6_, *δ* ppm): 8.56 – 8.42 (m, 3H), 7.87 (dd, *J* = 5.1, 4.3 Hz, 1H), 7.75 (d, *J* = 4.6 Hz, 1H), 7.57 – 7.45 (m, 4H), 7.30 (dd, *J* = 5.2, 1.9 Hz, 4H), 7.15 (d, *J* = 5.4 Hz, 2H), 6.81 – 6.76 (m, 3H), 5.87 (d, *J* = 10.6, 0.8 Hz, 1H), 5.54 (s, 2H), 5.30 (dd, *J* = 6.5, 0.6 Hz, 1H), 5.19 (s, 2H). Mass (*m/z*) calculated for C_33_H_24_N_2_O_3_ [M + H]^+^ = 497.1820; observed = 497.1848.

**Synthesis of *tert*-butyl (4-(1,3-dioxo-2-(4-((4-vinylbenzyl)oxy)phenyl)-2,3-dihydro-1H-** **benzo[de]isoquinolin-6-yl)phenyl)carbamate (NDIST).** **C3** (500.0 mg, 0.8 mmol) was taken in a 250 mL round-bottom flask and dissolved in 75 mL dioxane equipped with a magnetic stir bar. The reaction mixture was then placed in an ice-water bath and di-*tert-*butyl dicarbamate (0.8 mmol) was added dropwise into the reaction mixture. The reaction was continued to stir for 24 h. After completion of the reaction, the solution was diluted with 300 mL of DCM. This organic layer was washed with water (5 × 200 mL) and brine solution (200 mL). The organic layer was dried over anhydrous Na_2_SO_4_ and purified using column chromatography with 30% (v/v) EtOAc in hexane mixture. Yield = 45%. NDIST was characterized by ^1^H NMR and ESI-MS analysis. ^1^H NMR (500 MHz, DMSO-*d*_6_, *δ* ppm): 9.65 (s, 1H), 8.58 – 8.47 (m, 2H), 8.36 (d, *J* = 5.1 Hz, 1H), 7.88 (d, *J* = 5.1 Hz, 1H), 7.81 (d, *J* = 4.5 Hz, 1H), 7.71 (d, *J* = 5.2 Hz, 2H), 7.59 – 7.43 (m, 6H), 7.31 (d, *J* = 5.3 Hz, 2H), 7.15 (d, *J* = 5.4 Hz, 2H), 6.77 (d, *J* = 10.6 Hz, 1H), 5.87 (d, *J* = 10.6 Hz, 1H) 5.29 (d, *J* = 6.5, Hz, 1H), 5.19 (s, 2H), 1.53 (s, 9H). ^13^C NMR (126 MHz, DMSO-*d*_6_, *δ* ppm): 162.59, 163.74, 157.18, 150.92, 138.88, 137.21, 137.18, 136.79, 134.44, 131.27, 130.61, 130.27, 129.61, 128.76, 129.00, 128.49, 128.45, 127.49, 127.39, 126.71, 124.60, 122.94, 120.86, 115.30, 118.94, 108.99, 105.06, 70.64, 45.39 35.65. Mass (*m*/*z*) calculated for C_38_H_32_N_2_O_5_ [M + H]^+^ = 597.2311; observed = 597.2307.

**Synthesis of BCP5 and BCP10 copolymers.** A typical procedure for the reversible addition-fragmentation chain transfer (RAFT) polymerization technique was followed [5]. For example, **BCP5** (Table **S1**) was synthesized by taking DMAEMA (250.0 mg, 1.6 mmol), NDIST (50. 0 mg, 83.8 μmol), CDP (6.8 mg, 16.8 μmol), AIBN (0.6 mg, 3.7 μmol, from a stock solution of AIBN in DMF), and 1.2 mL DMF in a 20 mL polymerization vial equipped with a magnetic stir bar. Next, the polymerization vial was purged with N_2_ for 10 min and placed in a preheated block at 70 °C for 24 h. The polymerization process was quenched by immersing the vial in ice-water bath and exposing it to air. The resultant copolymer was precipitated in cold hexane, then dissolved in acetone and reprecipitated (4 times) from hexane. The polymers were purified by dialyzing against methanol using a membrane with a molecular weight cutoff (MWCO) of 6000-8000 Da. The methanol was replaced every 2 to 6 h for at least 5 times. The polymer was dried under vacuum for 12 h at 40 °C to obtain a yellow solid powder. Additionally, another copolymer, **BCP10**, was prepared following a similar procedure by varying the feed ratio of DMAEMA and NDIST.

**Boc groups deprotection from polymers.** The Boc groups of the copolymers were deprotected by using TFA in DCM at room temperature. Typically, in a 20 mL glass vial containing a small magnetic stir bar, 500 mg of copolymer was dissolved in 1 mL DCM. After that, 2.0 mL of TFA was dropwise added to the polymer solution and stirred for 4 h at room temperature. The polymer was then purified by precipitating in diethyl ether. The desired deprotected copolymer was dried in vacuum for 12 h to obtain an orange powder. The Boc group deprotected **BCP5** and **BCP10** were named as **DCP5** and **DCP10**, respectively.

**Limit of detection (LOD) determination**. Fluorescence titration measurements were carried out to determine the LOD value of **DCP5**. 1.0 mg/mL solution of **DCP5** was prepared in phosphate buffer saline (PBS) as a stock solution. Formaldehyde (FA) stock solution was prepared in Milli-Q water (freshly prepared by diluting 3.7 µL of commercial 37 wt. % FA solution in 500 µL of Milli-Q water). Different concentrations of FA (10-100 nM) were added to the **DCP5** solution and the fluorescence intensities were measured at 580 nm (excitation wavelength, *λ*_ex_ = 420 nm). The experiment was three times repeated to determine the standard deviation (*σ*) of the blank polymer samples. The LOD value was calculated using equation (1), following a well-established literature protocol [6].

$LOD= \frac{3\sigma}{K}$ (1)

where *K* represents the calibration curve slope at low FA concentrations, and *σ* denotes the standard deviation of the emission intensity for the blank polymer solution. The calculated LOD was 1.36 nM while, *K* = 4975 and *σ* = 2260.

**Density functional theory (DFT) calculation.** The geometry of the naphthalimide-conjugated repeating unit in the deprotected copolymer and the corresponding imine after the reaction with FA *via* addition-elimination reaction were optimized by DFT calculations. The calculations were evaluated with B3LYP functional and 6-311G as basis set using Gaussian 09 [7].

**Quantum yields measurements.** Absolute quantum yields were measured *via* an integrating sphere (Xe-400 lamp and PMT-900 + integrating sphere detector) method from Edinburgh Instruments.

**Synthesis of the benzophenone-based copolymers (P(DMAEMA-*co*-NDIST-*co-*BPMA)).** DMAEMA (383.9 mg, 1.8 mmol), NDIST (81.0 mg, 0.14 mmol), BPMA (36.14 mg, 0.14 mmol), CDP (10.4 mg, 0.03 mmol), and AIBN (0.9 mg, 5.2 μmol, from a stock solution of AIBN in DMF) were dissolved in 2 mL DMF in a 20 mL septum-capped vial. The reaction mixture contains 5% of NDIST and 5% of BPMA, while the molar ratio of [CDP]:[AIBN] was 1:0.2. The solution was purged with N_2_ for 10 min and then placed in a preheated block at 70 °C for 24 h. The polymerization reaction was quenched by putting the vial in an ice-water bath and exposed to air. The viscous polymer solution was precipitated in cold hexane. It was dissolved in acetone and reprecipitated (4 times) from hexane. The polymer, P(DMAEMA-*co*-NDIST-*co-*BPMA), was dried under vacuum for 12 h at 40 °C to obtain a yellow solid powder (Scheme **S2**). Monomer conversion was determined as 70% (gravimetric analysis).

**Synthesis of P(DMAEMA-*co*-DNDIST-*co-*BPMA).** Boc groups in P(DMAEMA-*co*-NDIST-*co-*BPMA) were deprotected using TFA in DCM at room temperature. Typically, in a 20 mL glass vial equipped with a small magnetic stir bar, 200 mg of P(DMAEMA-*co*-NDIST-*co-*BPMA) was dissolved in 0.5 mL DCM and placed in an ice-water bath. Next, 1.0 mL TFA was added dropwise to the solution and the reaction mixture was stirred for 4 h at room temperature. The polymer was purified by precipitation in diethyl ether, and dried under vacuum for 12 h to obtain an orange colored powdered P(DMAEMA-*co*-DNDIST-*co*-BPMA).

**General procedure for light-induced immobilization of BPMA-containing polymer.** For covalent attachment of P(DMAEMA-*co*-DNDIST-*co*-BPMA) onto a filter paper, the copolymer P(DMAEMA-*co*-DNDIST-*co*-BPMA) was dissolved in MeOH at a concentration of 5 mg/mL. Subsequently, the desired-sized filter papers were soaked in the polymer solution by dip-coating (submerging for 20 s) and air-drying for 30 min. Next, the materials were exposed to 365 nm UV light for 30 min to induce crosslinking of the polymers. Of note, to generate well-defined polymer patterns on the paper surface, black paper masks were placed on top of the polymer-coated filter paper during the illumination step.

**[Insert Scheme S1]**

**[Insert Figure S1]**

**[Insert Figure S2]**

**[Insert Figure S3]**

**[Insert Figure S4]**

**[Insert Figure S5]**

**[Insert Figure S6]**

**[Insert Table S1]**

**[Insert Figure S7]**

**[Insert Figure S8]**

**[Insert Figure S9]**

**[Insert Figure S10]**

**[Insert Figure S11]**

**[Insert Figure S12]**

**[Insert Figure S13]**

**[Insert Figure S14]**

**[Insert Scheme S2]**

**References**

[] Moad G, Chong YK, Postma A, et al. Advances in RAFT polymerization: the synthesis of polymers with defined end-groups. Polymer. 2005;46:8458-8468.

[2] Böhm A, Trosien S, Avrutina O, Kolmar H, Biesalski M. Covalent attachment of enzymes to paper fibers for paper-based analytical devices. Front. Chem. 2018;6:214 (1-10).

[3] Furniss BS, Hannaford AJ, Smith PDG, Tatchell AR, in Vogel’s Textbook of Practical Organic Chemistry, Longman Scientific & Technical Co-published in the United States with John Wiley & Sons, Inc., New York, 1989.

[4] Paul S, Ghosh S, Kumar M, et al. Water-soluble napthalimide-conjugated n-nitrosamine-based block copolymers for photoinduced nitric oxide delivery and cell imaging. ACS Appl. Polym. Mater. 2024;6:1075-1085.

[5] Das S, Banerjee A, Roy S, et al. Zwitterionic polysulfobetaine inhibits cancer cell migration owing to actin cytoskeleton dynamics. ACS Appl. Bio Mater. 2024, 7, 144-153.

[6] Zhu B, Gao C, Zhao Y, Liu C, et al. A 4-hydroxynaphthalimide-derived ratiometric fluorescent chemodosimeter for imaging palladium in living cells. Chem. Commun. 2011;47:8656-8658.

[7] Frisch MJ, Trucks GW, Schlegel HB, et al. In Gaussian 09, Gaussian, Inc, Wallingford, CT, USA, 2009.


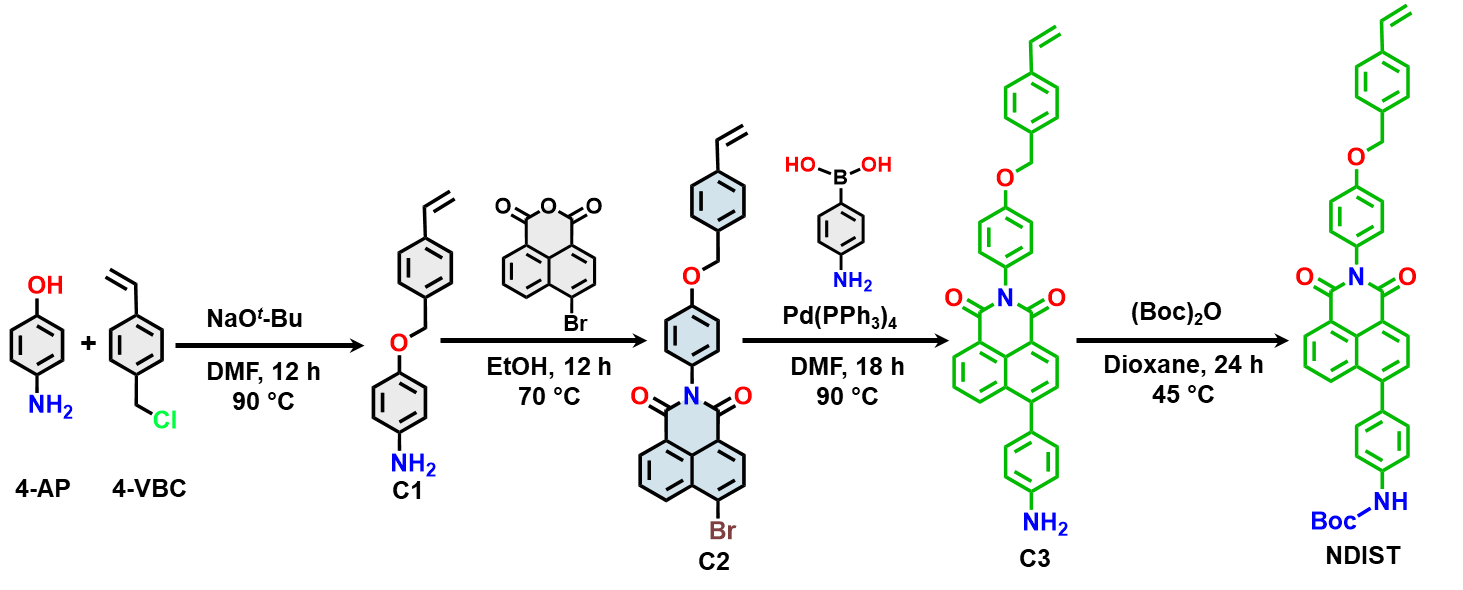


**Scheme S1.** Synthetic scheme for the monomer.


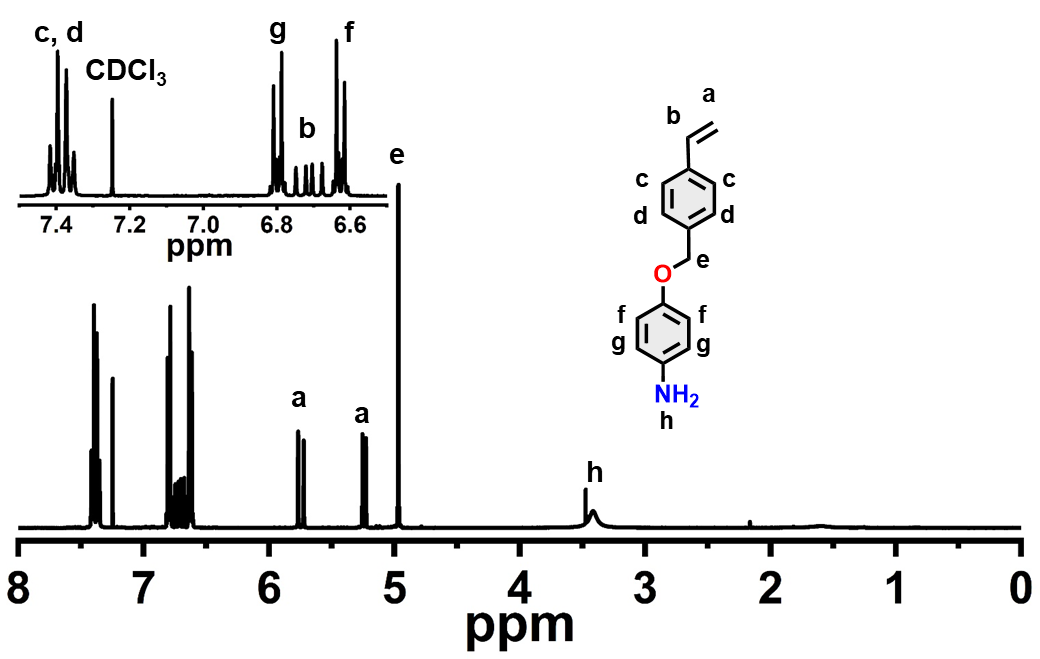


**Figure S1.** ^1^H NMR spectrum of **C1** recorded in CDCl_3_.


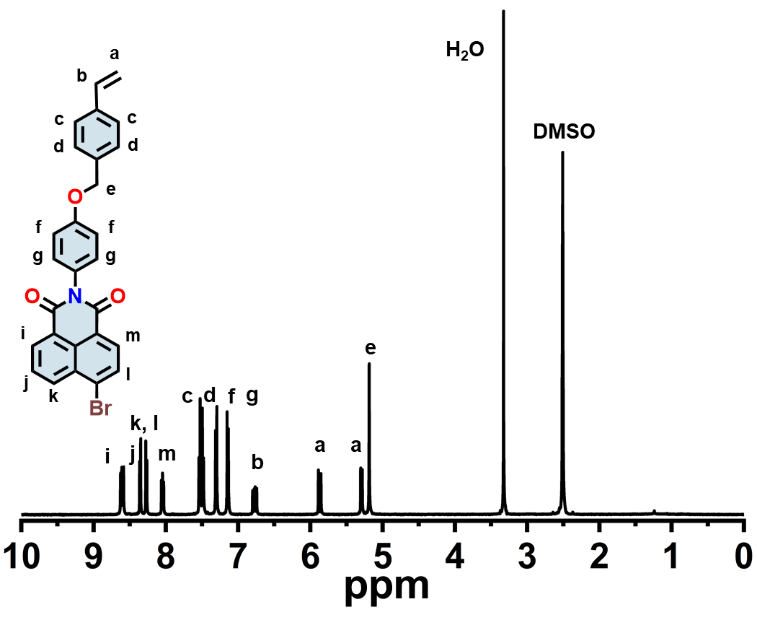


**Figure S2.** ^1^H NMR spectrum of **C2** recorded in DMSO-*d*_6_.


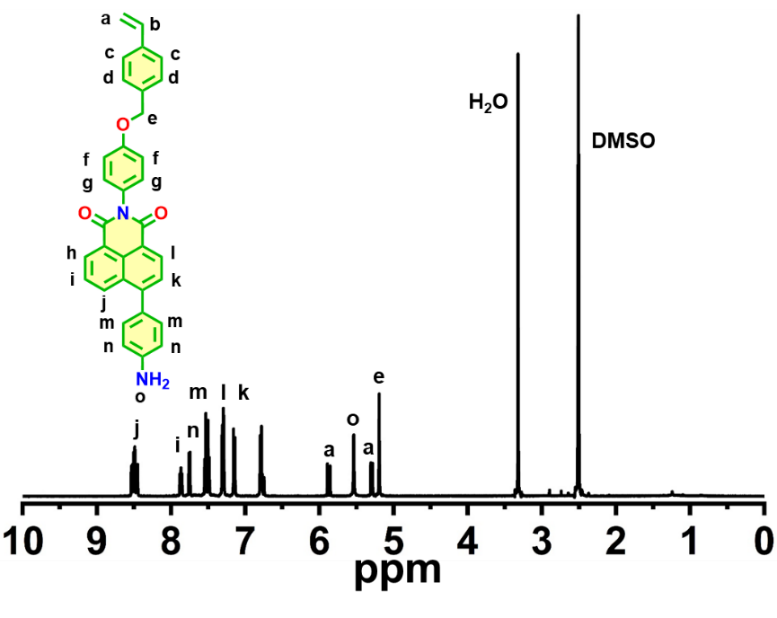


**Figure S3.** ^1^H NMR spectrum of **C3** in DMSO-*d*_6_.


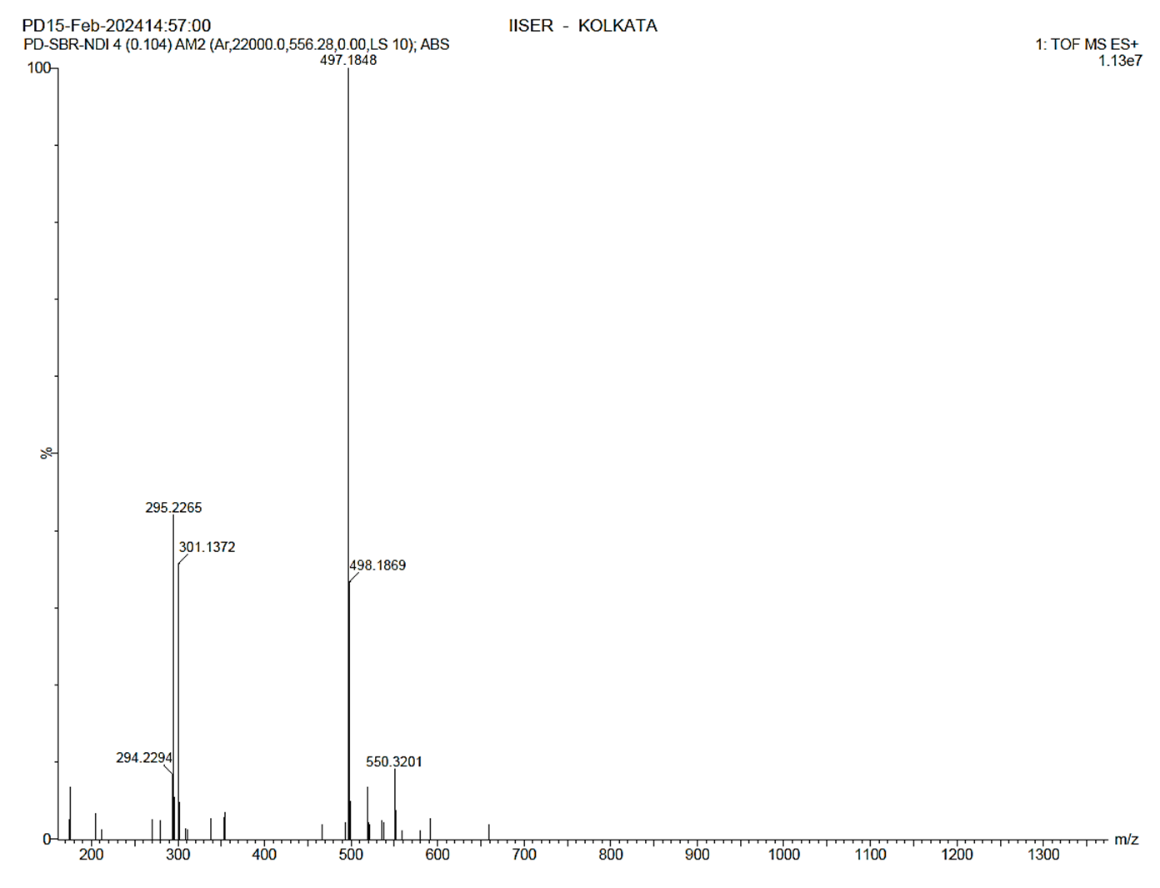


**Figure S4.** ESI-MS spectrum of **C3**. Mass (*m/z*) calculated for C_33_H_24_N_2_O_3_ [M + H]^+^ = 497.1820; observed = 497.1848.


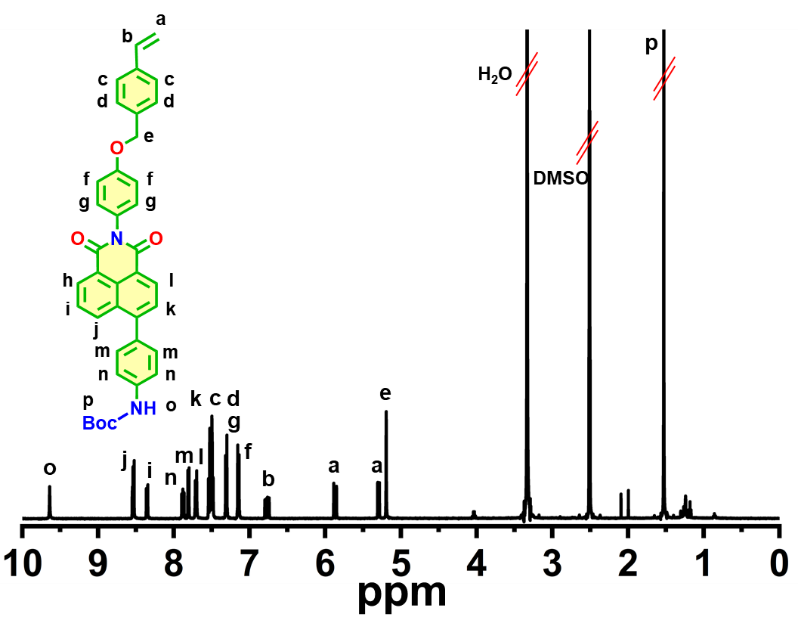


**Figure S5.** ^1^H NMR spectrum of **NDIST** in DMSO-*d*_6_.


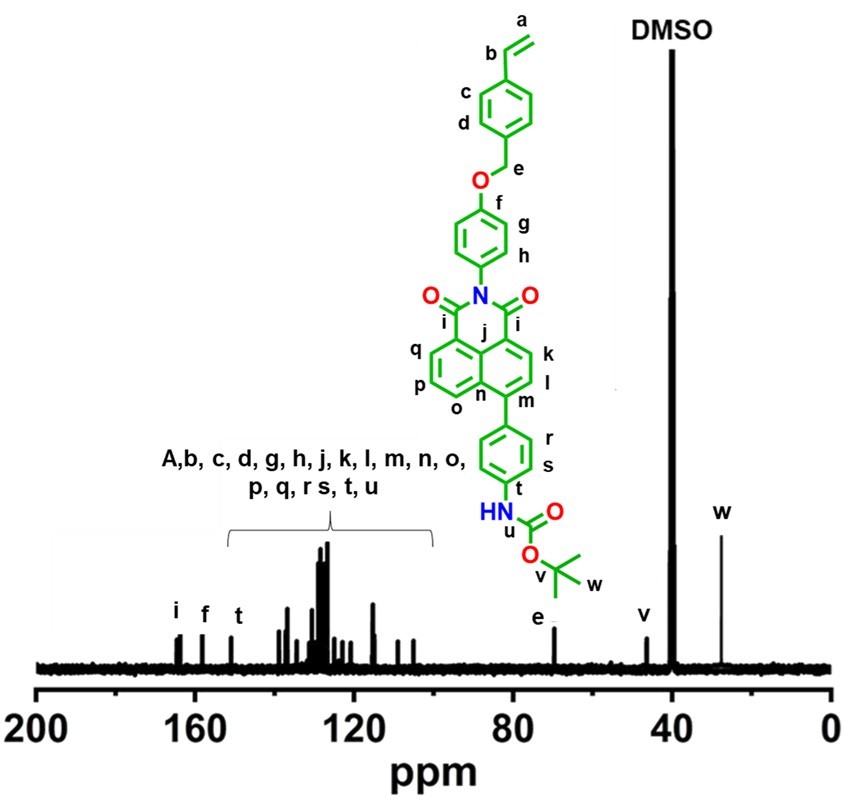


**Figure S6.** ^13^C NMR spectrum of **NDIST** in DMSO-*d*_6_.


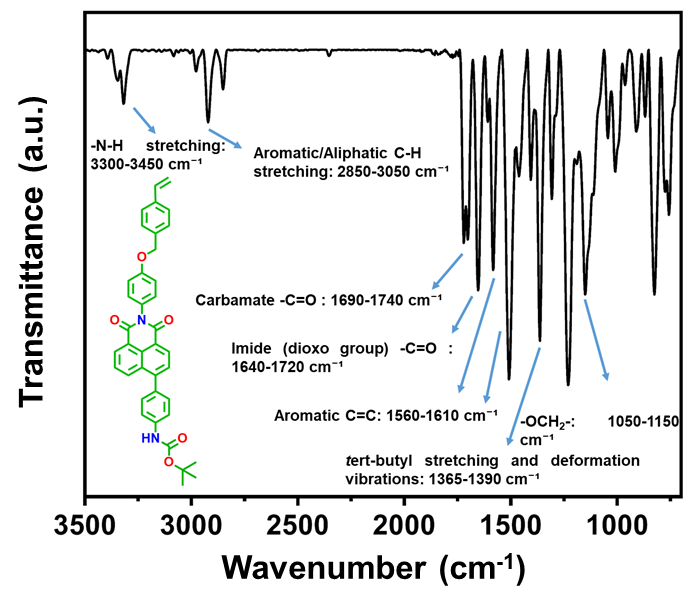


**Figure S7.** ATR-FTIR spectrum of monomer **NDIST**.


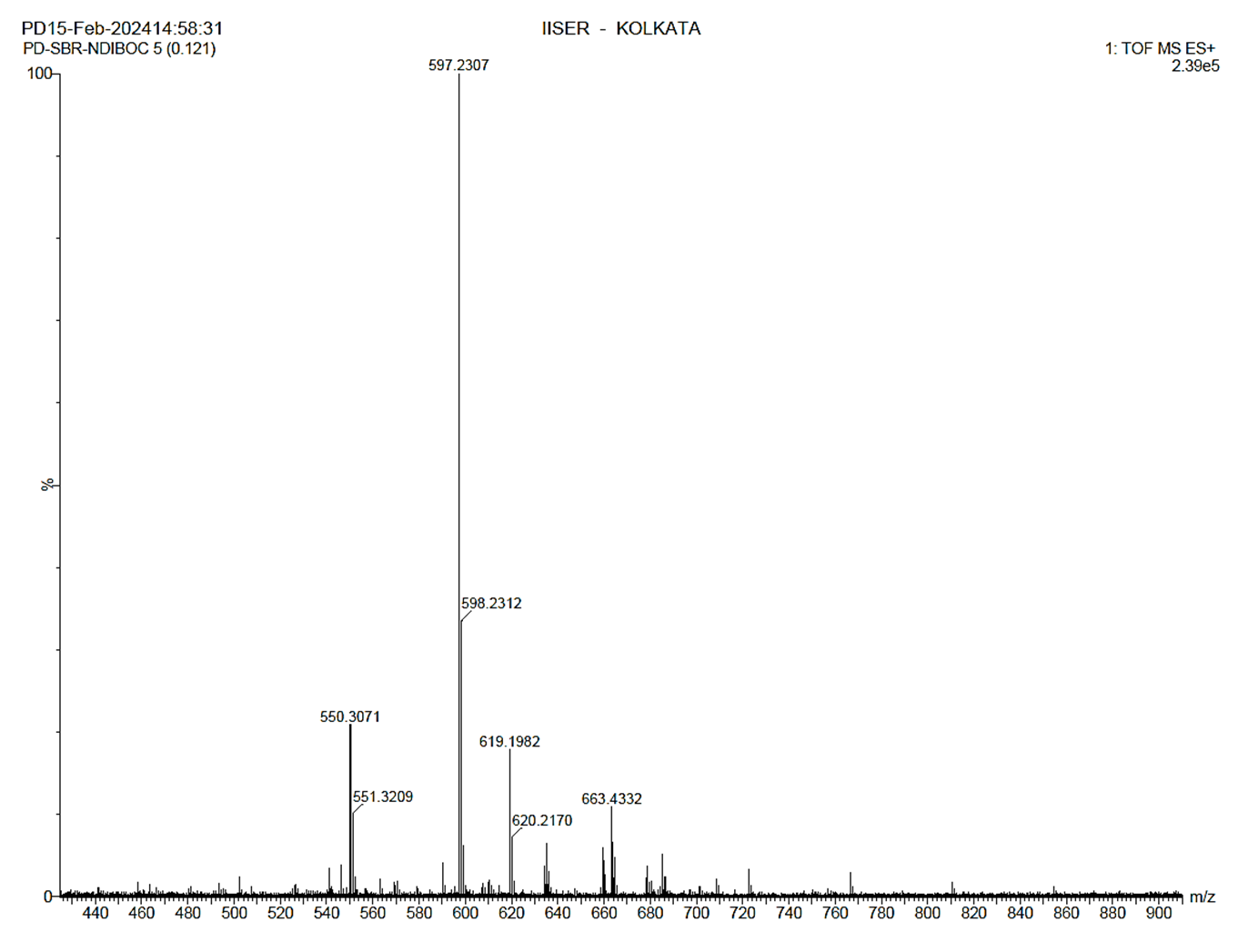


**Figure S8.** ESI-MS spectrum of NDIST. Mass (*m/z*) calculated for C_38_H_32_N_2_O_5_ [M + H]^+^ = 597.2311; observed = 597.2307.

**
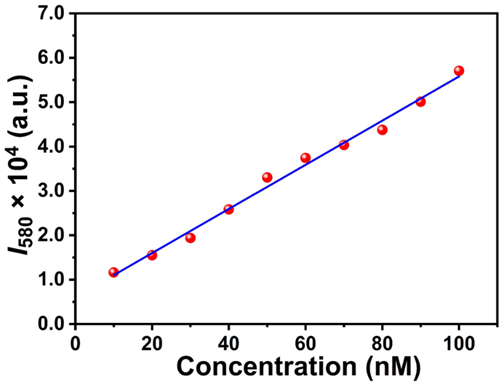
**

**Figure S9.** LOD calculation plot of **DCP5** in PBS.


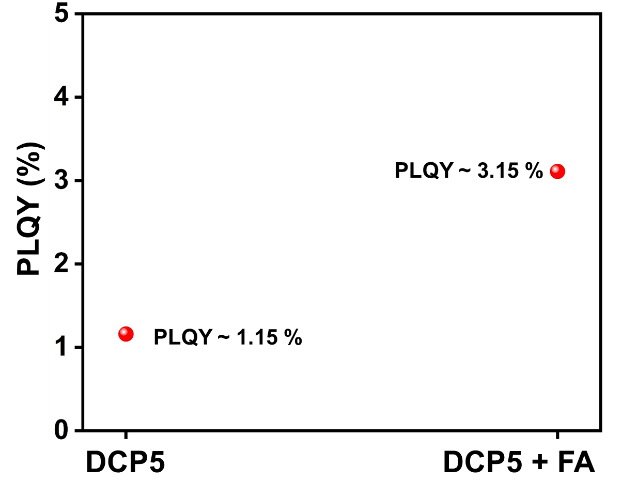


**Figure S10.** Photoluminescence quantum yield (PLQY) before and after formaldehyde addition to **DCP5** polymer in aqueous phase.


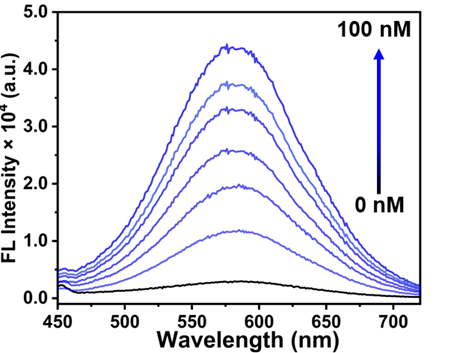


**Figure S11.** The emission spectra of **C3** in a DMSO/H_2_O mixture (2:8, v/v) (10^-4^ M) in the presence of various concentrations of FA (10 to 100 nM). Spectra were measured after 1 min after the FA addition.


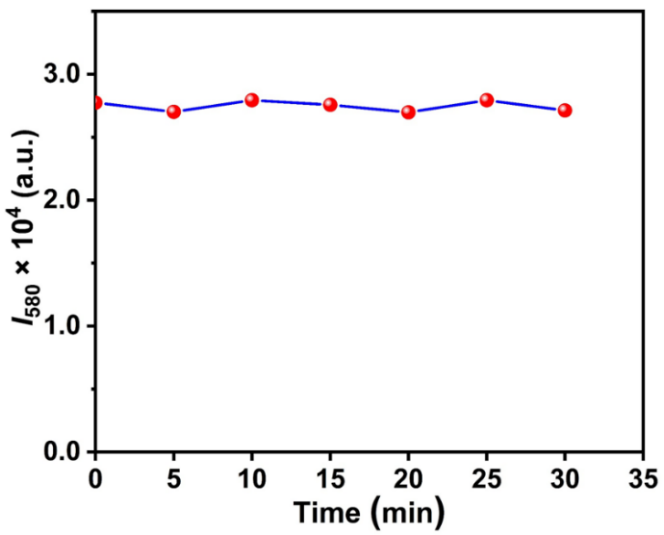


**Figure S12.** Photostability of the polymeric probe (**DCP5**) at 580 nm emission intensity (irradiation at 420 nm).


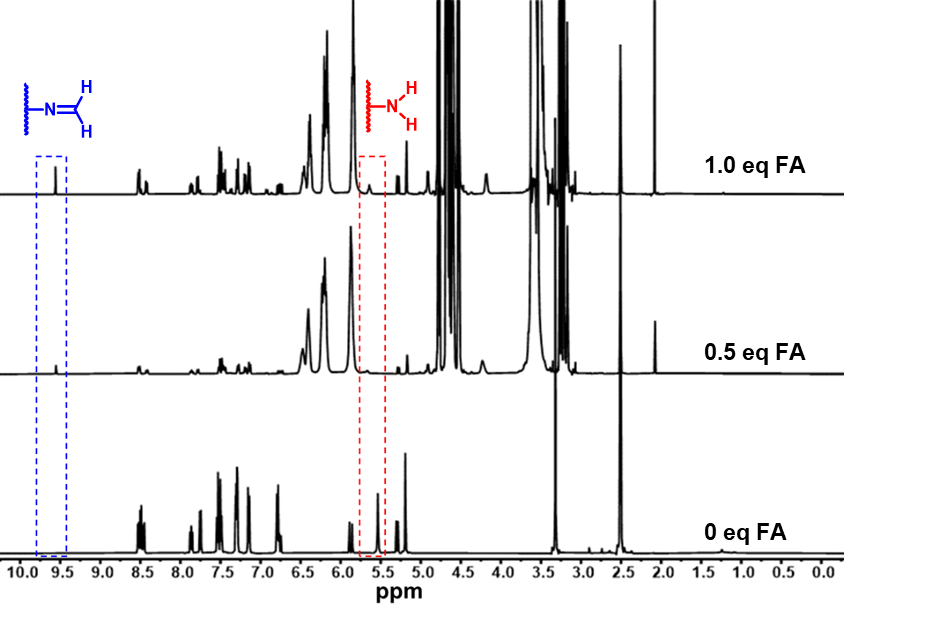


**Figure S13.** ^1^H NMR titration spectra in DMSO-*d*_6_ of the model compound **C3** with the increasing FA concentrations.


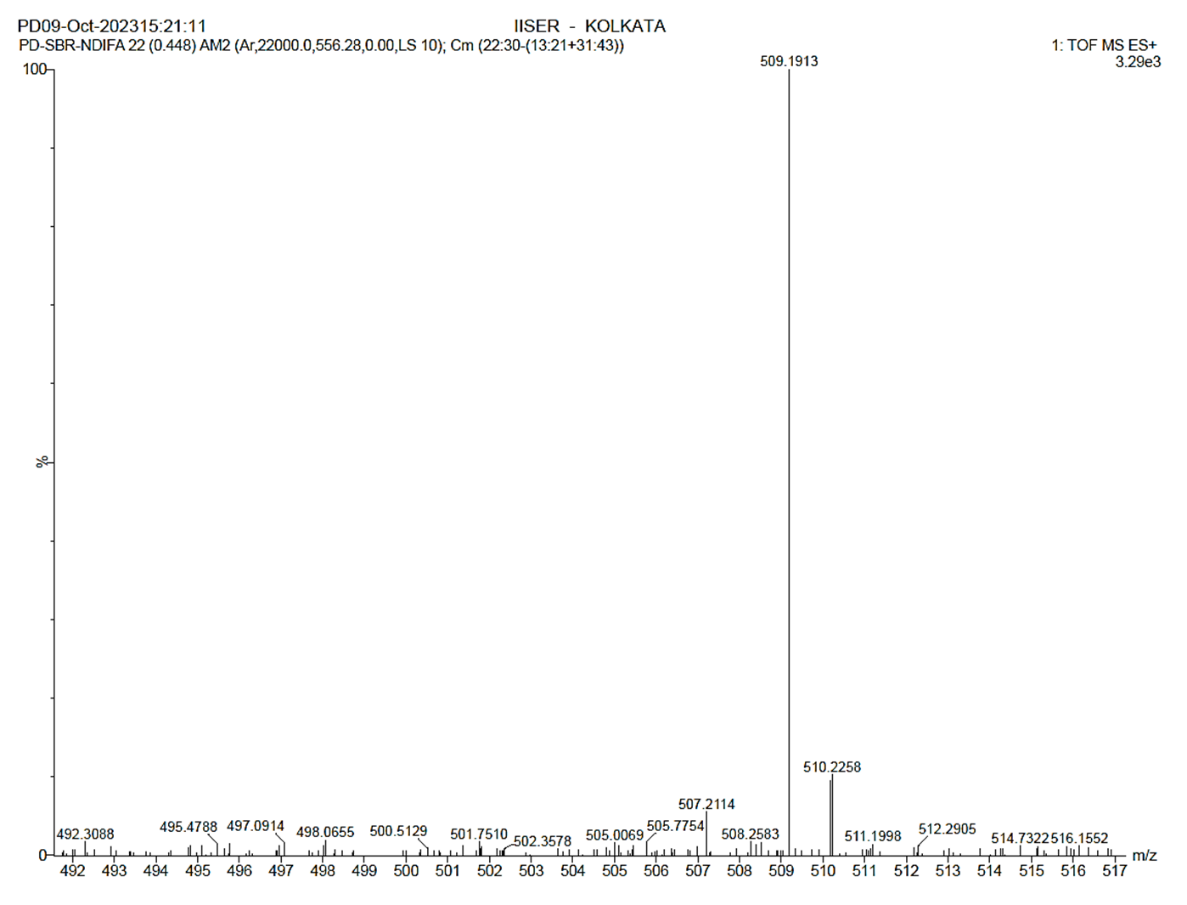


**Figure S14.** ESI-MS spectrum of **C3** in the presence of FA. Mass (*m/z*) calculated for C_34_H_24_N_2_O_3_ [M + H]^+^ = 509.1820; observed = 509.1913.


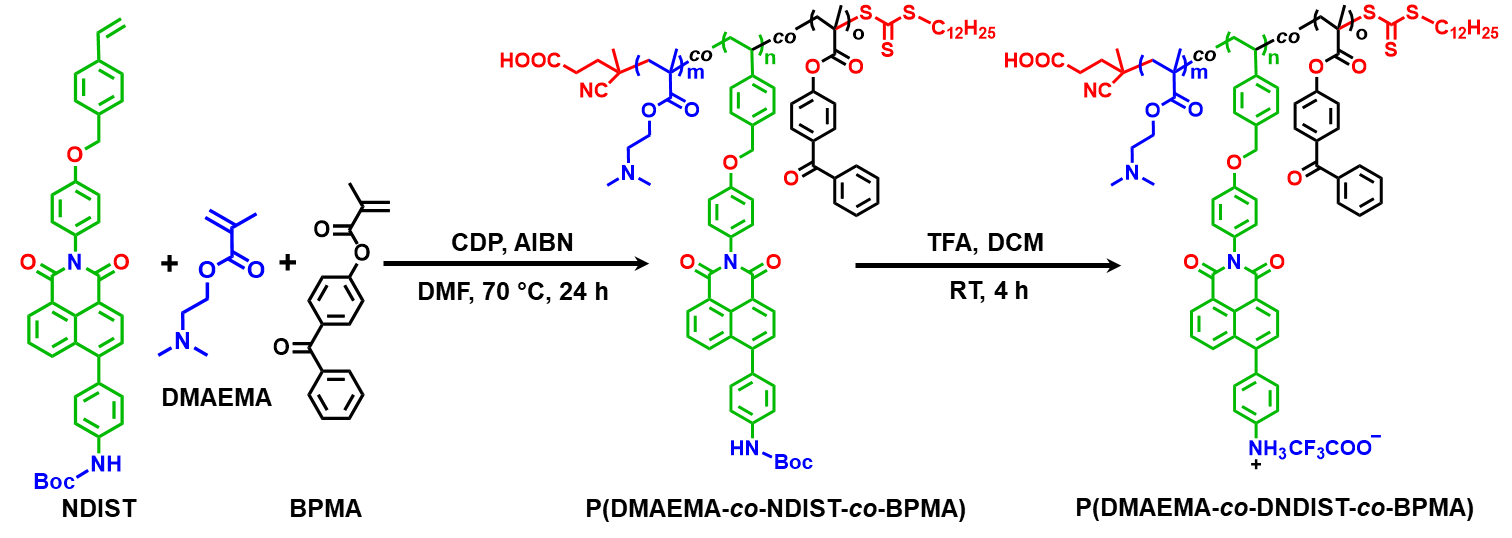


**Scheme S2.** Synthetic scheme for the preparation of BPMA-containing fluorescent polymeric probe.

**Table S1.** Characterization of different copolymers synthesized at 70 °C in DMF.

| Polymer | % of NDIST in feed | Conv.^b^ (%) | % of NDIST in copolymer^c^ | *M*_n,theo_^c^ (g/mol) | *M*_n,NMR_^d^  (g/mol) | *M*_n,SEC_^e^  (g/mol) | *Ɖ*^e^ |
| --- | --- | --- | --- | --- | --- | --- | --- |
| **BCP5^a^** | 5 | 70 | 4 | 12250 | 14900 | 12200 | 1.21 |
| **BCP10^a^** | 10 | 64 | 7 | 13100 | 17000 | 13600 | 1.18 |

^a^[Monomers]/[CDP]/[AIBN] = 100:1:0.2. For all the reactions, time = 24 h. ^b^Conversion (Conv.) was determined by gravimetric analysis. ^c^*M*_n,theo_ = ([Monomers]/[CDP] × (molecular weight (*MW*) of monomer) × Conv. + *MW* of CDP), where *MW* of monomer = average molecular weight considering feed mol% of monomers. ^d^Calculated from ^1^H NMR analysis. ^e^Obtained by SEC analysis.
